# The Giant Magnet as an Eye Preserver

**Published:** 1906-07

**Authors:** Ross P. Cox

**Affiliations:** Rome, Ga.


### THE GIANT MAGNET AS AN EYE PRESERVER.

BY ROSS P. COX, M.D.,

ROME, GA.

My first experience with magnetic aid in removing steel and iron from the eyeball was with the very large electromagnet used in the clinic of Prof. Fuchs, in Vienna, 1896-1897. This magnet was nearly as large as half a barrel and contained revolving machinery that made a great noise when in use.

I was so favorably impressed with some of Professor Fuchs' results than on returning I called on a big electric house in New York and discussed the magnet question with them. These people, June, 1897, dampened my enthusiasm considerably by asserting that under magnetism all particles would come out of the eye sidewise, and not endwise, and thus so much damage would arise that much more harm than good would be the net result.

That this supposition was incorrect was subsequently shown by my own experience.

However, I procured a light hand-magnet, operated by a small fluid cell. With this I succeeded only in removing a few bits of steel that were very superficially located and free from entanglements.

A few years later, encouraged by the published results of Haab, Wright, Sweet and others, I purchased an imported 70-pound instrument, about ten inches long and seven in diameter. It was operated by the 110-volt current, which, of course, was the direct or unbroken circuit, as no other kind will work a magnet.

This instrument did better, enabling me to extract iron fragments that had passed the cornea or sclera in about half the cases.

After a while, however, a man came to me with a bit of steel located near the center of his right lens. It had been there for about five months, and this magnet failed to dislodge it.

I then took up the matter again with Truax, Greene and Company, and had them make me a very much more powerful instrument, longer and heavier than the old one, and operated by the 500-volt direct current. This magnet will sustain a weight of more than 500 pounds. I have used it in seven intrabulbar cases with only one failure to get the steel.

This one failure was in a patient that came several days after the accident; there was cloudiness of the lens and failure to locate the foreign body. It was probably firmly anchored in the back of the ball. The patient declined excision and I have not seen him since.

In two of the six instances of successful removal, the steel was visible to the anterior chamber, attached to the iris and the removal was accomplished easily through a small peripheral corneal incision. Before the use of magnetism the operation in these two cases would probably have been accompanied by an iridectomy. The recovery of each was complete and without incident.

Of the four cases of deeper extraction, three gave best results, and one a rather bad one, so far as sight was concerned. This last patient came immediately after the accident. The steel was plainly visible, attached to the upper, outer quadrant of the optic disc. It had entered through the sclera, about one centimeter internally from the sclero-corneal junction. Its removal was effected in less than two minutes, through the original wound, slightly enlarged by a very keen Grefe cataract knife. There must have been considerable infection, as the reaction amounted almost to a panophthalmitis. Patient declined incision and one month later the vision of affected eye amounted to little more than light perception.

In the second case, an adult man, the steel was about the size of two pin heads. It entered more than one c. m. externally to corneal border, and he came the day after accident. Media were clear, but steel was not located until removal, which was accomplished in less than half a minute, through slightly enlarged original wound. It had probably penetrated just deep enough to be out of sight. The result was quick and complete recovery. It is in just such cases as this that a properly used magnet means a saved eye.

In the third case, of deeper extraction, the steel penetrated cornea externally, almost at the scleral junction. It cleared the iris, and was probably lodged in or near the ciliary body, as search of the fundus failed to show any trace of it.

The magnet was applied half-way between wound of entrance and center of cornea, and the steel entered the anterior chamber in a very short time, causing much pain in the process. Iritis lasted for over two weeks, but when last seen, four weeks after accident, vision was normal. This steel, a bit from a hoe, striking a rock, was half as large again as an elongated pin head.

The piece of steel in the fourth deeper case penetrated cornea, iris and lens in upper, inner quadrant. The patient is a boy fourteen years of age. The accident happened two weeks before patient came to me. The lens was densely clouded and body could not be located. Application of the magnet to the center of cornea caused the little rod, 3mm. long and 1 mm. in diameter, to enter the anterior chamber endwise, and not sidewise, for I witnessed the process distinctly. Its complete removal was effected, as usually, by applying the magnet tip to a small inferior peripheral corneal incision. There is a pretty strong adhesion between iris and lens capsule at point of original injury. After reaction had subsided I did a discission of the traumatic cataract.

This case is complicated by the old adhesion, and is too recent to give final result, but promises useful vision.

The enormous increase of steel and iron working industries has rendered the accidental introduction of fragments within the eyeball a relatively frequent occurrence.

Furthermore, much of the cultivated land, certainly that of North Georgia, is full of rocks. A bit of steel from a hoe, or other implement, to my knowledge has been a rather frequent cause of the loss of one and, occasionally, of both eyes.

It surely ought to be very earnestly impressed upon the minds and consciences of physicians to see that each sufferer has the benefit of this simple, safe and usually very efficient little operation. I believe that the most powerful magnet obtainable is the best. While the possibility of doing damage must never be lost sight of, experience seems to prove that the intelligent use of the strongest instrument will average the best results.

While it is always highly desirable to locate the steel before operating, this is often impossible, nor does the failure to do so prevent the best results in many cases.

If original wound has healed and the lens has escaped injury, I would counsel removal through new incision, made wherever extraction will be best facilitated and important structures most conserved. This will frequently lead to extraction through sclera, whether the case be fresh or old.

When once the fragment is in the anterior chamber, unless the corneal wound is fresh and ample, the removal is, of course, to be completed by applying the tip of magnet to a new peripheral corneal incision, which will usually be made inferiorily.

In making or enlarging scleral wounds, only the sharpest Grefe cataract knives should be used.

